# Einfluss der Quadrizepsmuskulatur auf den patellofemoralen Kontaktmechanismus bei Patienten mit strecknaher patellofemoraler Instabilität nach MPFL-Rekonstruktion

**DOI:** 10.1007/s00132-023-04413-2

**Published:** 2023-08-11

**Authors:** Markus Siegel, Elham Taghizadeh, Andreas Fuchs, Philipp Maier, Hagen Schmal, Thomas Lange, Tayfun Yilmaz, Hans Meine, Kaywan Izadpanah

**Affiliations:** 1 Department of Orthopedic Surgery and Traumatology, Freiburg University Hospital, Albert Ludwigs University Freiburg, Hugstetter Straße 55, 79106 Freiburg, Deutschland; 2 Institute for Medical Image Computing, Fraunhofer MEVIS, Universitätsallee 29, 28359 Bremen, Deutschland; 3 Division of Medical Physics, Department of Diagnostic and Interventional Radiology, Medical Center – University of Freiburg, Faculty of Medicine, University of Freiburg, Killianstraße 5a, 79106 Freiburg, Deutschland; 4 Dep. Of Orthopedic Surgery, University Hospital Odense, Sdr. Boulevard 29, 5000 Odense, Dänemark

**Keywords:** Knorpel, Gelenkinstabilität, Ligamente, Magnetresonanztomographie, Prospektive Studie, Cartilage, Joint instability, Ligaments, Magnetic resonance imaging, Prospective studies

## Abstract

**Einleitung:**

Die MPFL-Rekonstruktion stellt eine der wichtigsten operativen Therapiemöglichkeiten beim Auftreten rezidivierender Patellaluxationen bei strecknaher patellofemoraler Instabilität dar. Dennoch ist die Rolle der Quadrizepsmuskulatur bei Patienten mit patellofemoraler Instabilität vor und nach einer patellofemoralen Stabilisierung mittels MPFL-Plastik bislang nicht vollständig geklärt. Die vorliegende Studie untersucht den Einfluss der Quadrizepsmuskulatur auf den patellofemoralen Kontaktmechanismus bei Patienten mit strecknaher patellofemoraler Instabilität (PFI) vor und nach einer operativen patellofemoralen Stabilisierung mittels MPFL-Plastik anhand von statisch-dynamischen 3‑Tesla MRT-Datensätzen in frühen Beugegraden (0–30°).

**Methoden:**

In dieser prospektiven Kohortenstudie wurden 15 Patienten mit strecknaher PFI vor und 11 ± 5 Wochen nach einer isolierten MPFL-Rekonstruktion und 15 Probanden mit gesunden Kniegelenken mittels dynamischen MRT-Scans untersucht. Die MRT-Scans wurden in einer individuell angefertigten pneumatischen Kniebelastungsvorrichtung zur Bestimmung der patellofemoralen Knorpelkontaktfläche (CCA) mit und ohne Quadrizepsaktivierung (50 N axiale Last) durchgeführt. Vergleichende Messungen wurden anhand von 3D-Knorpel- und Knochen-Meshes in 0–30° Kniebeugung an den Patienten mit patellofemoraler Instabilität prä- und postoperativ und an den kniegesunden Probanden durchgeführt.

**Ergebnisse:**

Die präoperativ ermittelte patellofemorale CCA von Patienten mit strecknaher PFI betrug 67,3 ± 47,3 mm^2^ in 0°-Flexion, 118,9 ± 56,6 mm^2^ in 15°-Flexion und 267,6 ± 96,1 mm^2^ in 30°-Flexion. Unter aktivierter Quadrizepsmuskulatur (50 N) zeigte sich eine Kontaktfläche von 72,4 ± 45,9 mm^2^ in Streckung, 112,5 ± 54,9 mm^2^ in 15° Beugung und 286,1 ± 92,7 mm^2^ in 30° Beugung ohne statistische Signifikanz im Vergleich. Die postoperativ bestimmte CCA ergab in 0°, 15° und 30°-Flexion 159,3 ± 51,4 mm^2^, 189,6 ± 62,2 mm^2^ und 347,3 ± 52,1 mm^2^. Unter Quadrizepsaktivierung mit 50 N zeigte sich eine CCA in Streckung von 141,0 ± 63,8 mm^2^, 206,6 ± 67,7 mm^2^ in 15° und 353,5 ± 64,6 mm^2^ in 30° Beugung, ebenso ohne statistischen Unterschied im Vergleich zur unbelasteten CCA. Bei Probanden mit gesunden Kniegelenken zeigt sich bei 30° Beugung ein Zuwachs der CCA von 10,3 % (*p* = 0,003).

**Schlussfolgerung:**

Obwohl sich die patellofemorale CCA nach MPFL-Plastik bei Patienten mit strecknaher patellofemoraler Instabilität signifikant vergrößert, zeigt sich weder prä- noch postoperativ ein signifikanter Einfluss der Quadrizepsmuskulatur.

## Abstract (en)

**Introduction:**

MPFL reconstruction represents one of the most important surgical treatment options for recurrent patellar dislocations at low flexion angles associated with low flexion patellofemoral instability. Nevertheless, the role of quadriceps muscles in patients with patellofemoral instability before and after patellofemoral stabilization using MPFL reconstruction has not been fully elucidated. The present study investigates the influence of quadriceps muscles on the patellofemoral contact in patients with low flexion patellofemoral instability (PFI) before and after surgical patellofemoral stabilization using MPFL reconstruction using 3 T MRI datasets in early degrees of flexion (0–30°).

**Methods:**

In this prospective cohort study, 15 patients with low flexion PFI before and after MPFL reconstruction and 15 subjects with healthy knee joints were studied using dynamic MRI scans. MRI scans were performed in a custom-made pneumatic knee loading device to determine the patellofemoral cartilage contact area (CCA) with and without quadriceps activation (50 N). Comparative measurements were performed using 3D cartilage and bone meshes in 0–30° knee flexion in the patients with patellofemoral instability preoperatively and postoperatively.

**Results:**

The preoperative patellofemoral CCA of patients with low flexion PFI was 67.3 ± 47.3 mm^2^ in 0° flexion, 118.9 ± 56.6 mm^2^ in 15° flexion, and 267.6 ± 96.1 mm^2^ in 30° flexion. With activated quadriceps muscles (50 N), the contact area was 72.4 ± 45.9 mm^2^ in extension, 112.5 ± 54.9 mm^2^ in 15° flexion, and 286.1 ± 92.7 mm^2^ in 30° flexion without statistical significance. Postoperatively determined CCA revealed 159.3 ± 51.4 mm^2^ , 189.6 ± 62.2 mm^2^ and 347.3 ± 52.1 mm^2^ in 0°, 15° and 30° flexion. Quadriceps activation with 50 N showed a contact area in extension of 141.0 ± 63.8 mm^2^, 206.6 ± 67.7 mm^2^ in 15° flexion, and 353.5 ± 64.6 mm^2^ in 30° flexion, also without statistical difference compared with unloaded CCAs. Subjects with healthy knee joints showed an increase of 10.3% in CCA at 30° of flexion (*p* = 0.003).

**Conclusion:**

Although patellofemoral CCA increases significantly after isolated MPFL reconstruction in patients with low flexion patellofemoral instability, there is no significant influence of quadriceps muscles either preoperatively or postoperatively.

**Graphic abstract:**

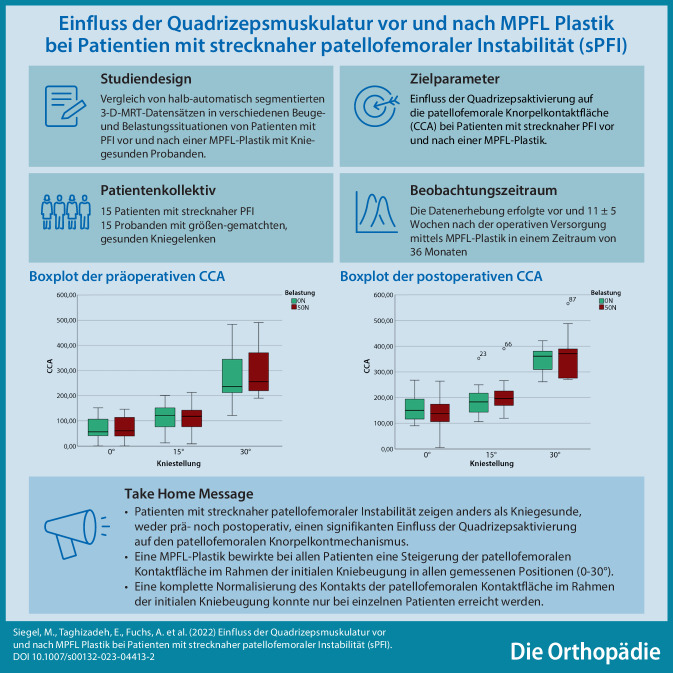

## Einleitung

Die patellofemorale Instabilität (PFI) wird als ein Zusammenspiel verschiedener Pathologien betrachtet, die in die knöcherne Geometrie, die stabilisierenden Weichteilstrukturen und in die dynamische Muskelaktivität unterteilt werden [1, 2]. Im frühen Flexionsbereich (0–30°) sind die passiven Weichteilstabilisatoren, wie das mediale patellofemorale Ligament (MPFL) oder das mediale patellotibiale Ligament (MPTA) sowie die Retinaculae, aber auch die aktive muskuläre Führung, mit dem größten Einfluss ab etwa 30° Beugung, für die Führung der Kniescheibe entscheidend [3–5]. Das MPFL liefert diesbezüglich etwa 50–60 % der gesamten Haltekräfte in den ersten 0–30° der Kniebeugung [6]. Daher wird die primäre Stabilisierung der Kniescheibe, bei strecknaher Flexionsinstabilität (0–30° Beugung) häufig, im Falle der Indikation zur operativen Versorgung und fehlender Begleitpathologien, über eine MPFL-Rekonstruktion durchgeführt [7]. Obwohl das Verfahren niedrige Rezidivraten und gute bis exzellente klinische Ergebnisse aufweist, ist wenig über die Auswirkungen der MPFL-Plastik auf den patellofemoralen Kontaktmechanismus in der frühen Kniebeugung bekannt [8–11]. Ebenso ist die Rolle der Quadrizepsmuskulatur als aktiver Stabilisator der Kniescheibe bei Patienten mit PFI vor und nach einer patellofemoralen Stabilisierung mittels MPFL-Plastik bislang nicht vollständig geklärt. Im Rahmen verschiedener In-vitro- als auch In-vivo-Studien konnten bisweilen Rückschlüsse über den patellofemoralen Kontakt in der frühen Beugung gezogen werden [12–14]. Kürzlich konnte gezeigt werden, dass patellofemoral stabilisierende Operationen, insbesondere mittels MPFL-Plastik, zu einer Zunahme der Knorpelkontaktfläche führen [14–16]. Bei kniegesunden Probanden steigt der Einfluss der Muskulatur, als aktiver Stabilisator, auf das patellofemorale Gelenk (PFJ) in den ersten 30° der Kniebeugung an und erreicht das Maximum bei etwa 30° Flexion [3]. Es wird vermutet, dass der stabilisierende Einfluss der muskulären, aktiven Stabilisatoren auf das patellofemorale Tracking bei Patienten, insbesondere bei strecknaher PFI, herabgesetzt ist, was mitunter den Grundstein für konservative Therapieansätze bildet [17, 18].

Die patellofemorale Knorpelkontaktfläche (CCA) wird als Parameter der patellofemoralen Kongruenz angesehen, dennoch ist die Bedeutung dieses Parameters bei Patienten mit PFI bisher noch nicht vollständig geklärt. Darüber hinaus fehlen weiterhin qualitativ hochwertige In-vivo-Daten darüber, wie sich die MPFL-Rekonstruktion auf die patellofemorale Kinematik auswirkt und insbesondere welchen Einfluss die Quadrizepsaktivität auf den patellofemoralen Kontaktmechanismus hat. Die vorliegende Studie untersucht den Einfluss der Quadrizepsaktivierung auf die Knorpelkontaktfläche bei kniegesunden Probanden und Patienten mit strecknaher PFI vor und nach einer patellofemoral stabilisierenden Operation mittels MPFL-Plastik in vivo anhand von hochauflösender 3‑Tesla-MRT-Datensätzen in verschiedenen Kniegelenksposition (0–30°).

Wir stellen die Hypothese auf, dass Patienten mit PFI gegenüber Probanden mit gesunden Kniegelenken sowohl vor als auch nach einer MPFL-Rekonstruktion keine signifikante Veränderung der patellofemoralen CCA bei Quadrizepsaktivierung aufweisen.

## Methoden

In der vorliegenden prospektiven gematchten Kohortenstudie wurden 15 Patienten mit strecknaher PFI und 15 Probanden mit gesunden Kniegelenken vor und nach einer MPFL-Rekonstruktion mittels dynamischen MRT-Scans untersucht. Die MRT-Scans wurden in einer speziell angefertigten Knieschiene mit einer MRT-gängigen pneumatischen Belastungsvorrichtung zur Bestimmung der patellofemoralen CCA in drei verschiedenen Beugepositionen (0°, 15° und 30°) durchgeführt. Vergleichende Messungen erfolgten anhand von 3‑D-Knorpel- und Knochen-Meshes.

Die Studie wurde vom Institutional Review Board (Ethikkommission der Universität Freiburg, ID 443/16) genehmigt und im Deutschen Studienregister (DRKS 00029213) eingetragen. Alle Probanden erteilten vor der Teilnahme das schriftliche Einverständnis. Alle Studienteilnehmer nahmen freiwillig und in Übereinstimmung mit der Erklärung von Helsinki an der Studie teil.

Die Einschlusskriterien für die Patienten waren eine symptomatische PFI bei niedriger Flexion (im Bereich von 0–30°) mit indizierter stabilisierender Operation durch eine MPFL-Rekonstruktion, Alter zwischen 18 und 65 Jahren ohne vorangegangene patellofemorale Gelenkoperation. Zu den Ausschlusskriterien gehörten stattgehabte patellofemorale Operationen, einliegendes Implantatmaterial aus früheren kniegelenksnahen Operationen, bestehende Schwangerschaft, retropatellare Arthrose und Klaustrophobie. Nur freiwillige Probanden ohne Knieschmerzen oder stattgehabte Knietraumata in der Vorgeschichte wurden in die Studie aufgenommen. Die Patienten wurden anhand der Warteliste für eine Operation mittels MPFL-Plastik ermittelt und telefonisch kontaktiert oder direkt in der ambulanten Sprechstunde rekrutiert. Die kniegesunden Probanden wurden über private Kontakte, über unsere Ambulanz oder über eine öffentliche Ausschreibung rekrutiert.

Die PFI wurde durch eine detaillierte klinische Untersuchung, a‑p-Röntgenaufnahmen der unteren Extremität (Beinganzaufnahme) und axiale Patellaaufnahmen sowie diagnostische MRT-Scans diagnostiziert.

Es konnten insgesamt 20 Patienten mit strecknaher PFI und 21 Volontäre mit gesunden Kniegelenken ermittelt, für diese Studie rekrutiert und mit dem MRT-Protokoll untersucht werden. Drei der präoperativen Messungen mussten aufgrund von technischen Problemen und einer nicht diagnostizierten Klaustrophobie beendet werden und konnten nicht rechtzeitig vor dem geplanten chirurgischen Eingriff wiederholt werden. 17 Patientenscans waren folglich für die postoperative Messung geeignet. Ein Patient entschied sich jedoch gegen einen chirurgischen Eingriff, und eine weitere Messung konnte wegen schwerer Artefakte nicht für die weitere Analyse berücksichtigt werden, sodass insgesamt 15 postoperative Aufzeichnungen zur Verfügung standen.

**Figure 1:**
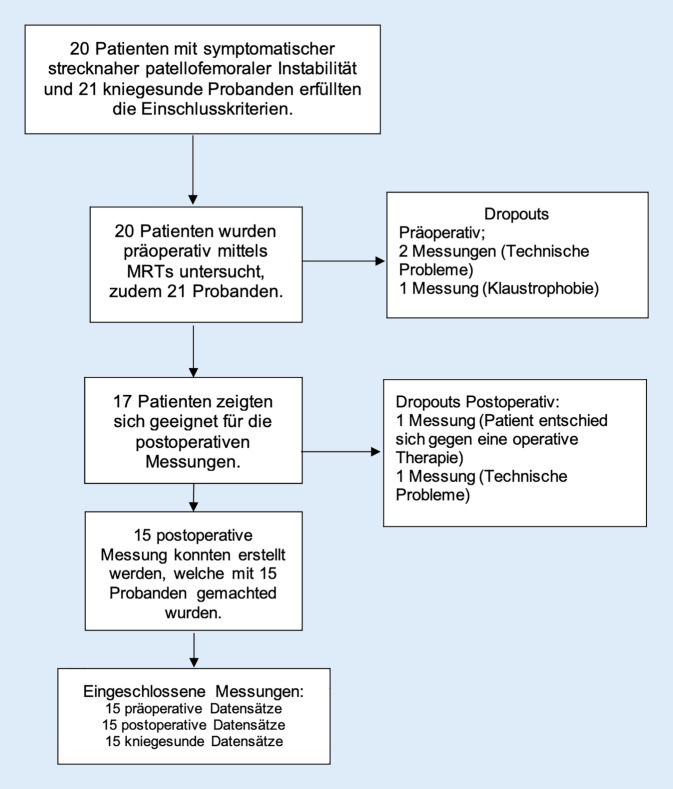

Folglich konnten 15 Patienten mit prä- und postoperativen Datensätzen für die weitere Analyse in die Studie aufgenommen und mittels TEA und Geschlecht mit 15 Probanden mit gesunden Kniegelenken gematched werden, wobei die 0°- und 15°-Messung eines postoperativen Patienten aufgrund eines ausgeprägten Gelenkergusses zudem exkludiert wurden (Abb. 1).

### Studienteilnehmer

Eine Übersicht über die Merkmale der Studienteilnehmer ist in Tab. 1 zu finden.

**Table 1:** 

	Patienten	±SD	Kontrollgruppe	±SD	
M, Anzahl	15	–	15	–	–
Alter (Jahre)	27,7	±7,5	30,3	±6,2	–
BMI (kg/m^2^)	20,7	±2,5	23,0	±1,8	–
Größe (cm)	173,3	±9,1	174,9	±8,2	–
Gewicht (kg)	71,5	±9,1	70,5	±8,1	–
Seite (rechts/links)	8/7	–	7/8	–	–
Geschlecht (weiblich/männlich)	9/6	–	9/6	–	–
TEA-Abstand (mm)	77	±6	78	±6	(*p* = 0,264)

*BMI* Body-Mass-Index, *TEA* transepikondylärer Abstand

Zur Matched-Pair-Analyse konnten die Studiengruppen mit einer gleichen Geschlechterverteilung von 9 weiblichen und 6 männlichen Teilnehmern generiert werden. Der transepikondyläre Abstand (TEA), als ein Parameter der Kniegröße, betrug bei den Pateinten mit PFI 77 ± 6 mm und 78 ± 6 mm bei den kniegesunden Voluntären und diente ebenfalls als „matching reference“ der beiden Kohorten (*p* = 0,264).

### Protokoll der MRT-Scans

Die MRT-Scans fanden präoperativ sowie 11 ± 5 Wochen nach der operativen Behandlung mittels MPFL-Plastik statt und wurden auf einem Magnetom Trio 3 T System (Siemens Healthineers, Erlangen, Deutschland) durchgeführt, wobei für den Signalempfang eine 8‑Kanal-Mehrzweckspule (NORAS MRI products, Höchberg) verwendet wurde, welche mit einem Klettverschluss am Oberschenkel befestigt wurde.

**Figure 2:**
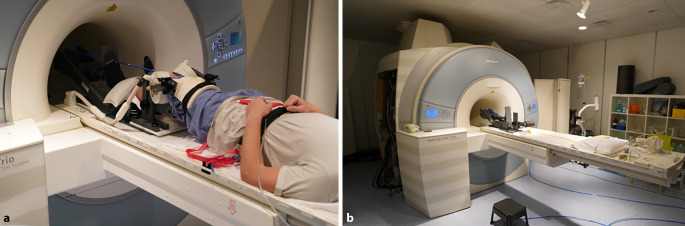

Um den MRT-Aufbau so reproduzierbar wie möglich zu gestalten, wurde die Versuchsperson mit einem Gewichthebergürtel am Scannerbett befestigt und der Fuß des gemessenen Beins in den Schlitten einer pneumatischen Kniebelastungsvorrichtung gelegt, die eine genaue Belastungseinstellung im Bereich von 0–500 N vom Kontrollraum aus ermöglicht [19]. Abb. 2 zeigt die pneumatische Belastungsvorrichtung und den MRT-Versuchsaufbau.

Als Sicherheitsvorkehrung erhielten die Probanden einen Notfallschalter, mit dem sie die Belastung sofort abschalten konnten.

Die Messungen erfolgten mit Kniebeugewinkeln von 0° (Extension), 15° und 30°. Bei den 0°- und 15°-Scans wurde das Knie mit Handtüchern abgestützt, während es bei der 30°-Messung mit einer schaumstoffgepolsterten Kniehalterung stabilisiert wurde. Die Messungen mit Erhebung der 3‑D-Datensätze wurden zunächst ohne und anschließend mit mechanischer Belastung von 50 N durchgeführt. Um eine Anpassung an die Belastung zu gewährleisten, wurden die MRT-Scans mit einer Verzögerung von 30 s nach Beginn der Belastung durchgeführt.

Für die MRT-Scans wurde ein 3D-Turbo-Spin-Echo(TSE)-Protokoll mit paralleler Bildgebung (GRAPPA mit Bildbeschleunigung um den Faktor 2) und einer isotropen Auflösung von 0,5 mm verwendet. Weitere Scanparameter waren: TR = 1,8 s, TE = 59 ms, Empfängerbandbreite = 504 Hz/Px, Scandauer = 6:20 min. Das 3‑D-Messvolumen wurde so positioniert, dass es das gesamte Patellofemoralgelenk abdeckte. Zur Abschwächung von Bewegungsartefakten wurde eine prospektive Bewegungskorrektur mit einem Moiré-Phase-Tracking(MPT)-System (Metria Innovation, Milwaukee, WI, USA) durchgeführt, welches aus einer einzelnen Kamera besteht, die an der Oberseite der Scannerbohrung angebracht ist, sowie einem einzelnen Tracking-Marker, der auf der Kniescheibe befestigt wurde [20–22].

Das Tracking-System erfasst Translations- und Rotationsbewegungen des Markers mit einer Rate von 80 Bildern pro Sekunde. Anhand der optisch verfolgten Bewegungsdaten wurde das MRT-Messvolumen vor jedem Anregungsimpuls in Echtzeit aktualisiert. Darüber hinaus wurde durch eine Bewegungskorrektur zwischen den Scans („position locking“) sichergestellt, dass alle MRT-Scans mit dem ursprünglich geplanten Sichtfeld (FOV) durchgeführt wurden.

Die Nachbearbeitung und Quantifizierung der Daten erfolgte mit der Bildverarbeitungsplattform SATORI, die von Fraunhofer MEVIS auf der Basis von MeVisLab entwickelt wurde. Die Segmentierung wurde zunächst manuell für die gesamte Probandenkohorte durchgeführt. Aus den segmentierten Strukturen wurden 3‑D-Netze der einzelnen Strukturen generiert, die dann mittels Bildregistrierung auf alle anderen Belastungs- und Beugesituationen (0°, 15° und 30° ohne bzw. mit 50 N Belastung) übertragen wurden. Die Bildregistrierung wurde für jeden Knochen einzeln durchgeführt, wobei ein normalisiertes Gradientenfeld-Abstandsmaß (NGF) auf den MRT-Daten optimiert wurde, das auf eine Region um den jeweiligen Knochen beschränkt war. Ausgehend von der Annahme, dass sich die Knochen nicht signifikant verformen, ist das Ergebnis dieser Knochenausrichtung eine individuelle starre Transformationsmatrix für jeden Knochen und somit Netzmodelle, die dann zur Analyse der CCA und der patellofemoralen Kinematik verwendet wurden.

Auf diese Weise wurden für jeden Studienteilnehmer in allen drei Beugestellungen die Knorpeloberflächen der Patella und des Oberschenkelknochens sowie die patellofemorale Knorpelkontaktfläche mit und ohne Aktivierung der Quadrizepsmuskulatur bestimmt [19, 20, 23].

Die Knorpelkontaktfläche wurde als der patellofemorale Knorpelbereich definiert, in dem der euklidische Abstand zwischen den beiden gegenüberliegenden Knorpelflächen weniger als 1 mm betrug [9]. Die postoperativen Messungen in Extension und in 15° Flexion eines Patienten konnten aufgrund eines postoperativen Gelenkergusses nicht auf diese Weise berechnet werden und wurden daher nicht in die weitere Analyse einbezogen.

### Statistische Methoden

Die deskriptiven Statistiken wurden in Form von Mittelwerten und Standardabweichungen angegeben. Die Unterschiede zwischen den verschiedenen Untergruppen wurden mittels Wilcoxon-Vorzeichen-Rang-Test analysiert. Ein *p*-Wert von weniger als 0,05 wurde als statistisch signifikant angesehen.

Die statistischen Analysen wurden mit IBM SPSS Statistics Version 28.0.0.0 (IBM Corp., Armonk, NY, USA) durchgeführt. Die Ergebnisse aller statistischen Tests wurden in einem explorativen Sinne interpretiert. Die *p*-Werte und 95 %-Konfidenzintervalle wurden folglich nicht für Mehrfachvergleiche korrigiert und die daraus gezogenen Schlussfolgerungen sind möglicherweise nicht reproduzierbar.

## Ergebnisse

Insgesamt konnten die Datensätze von 15 Patienten mit strecknaher PFI vor und nach einer MPFL-Rekonstruktion und von 15 Probanden mit gesunden Kniegelenken eingeschlossen werden.

### Quantifizierung der Knorpeloberfläche

Die präoperative Auswertung der Knorpelfläche von Patienten mit PFI ergab eine mittlere femorale Knorpelfläche von 68,4 ± 14,5 cm^2^. Die mittlere Knorpeloberfläche der Patella betrug 14,2 ± 4,2 cm^2^.

Probanden mit gesunden Kniegelenken zeigte eine durchschnittliche femorale Knorpeloberfläche von 71,1 ± 12,6 cm^2^ sowie eine patellare Knorpeloberfläche von 16,5 ± 2,2 cm^2.^

### Knorpelkontaktfläche (CCA)

#### Patienten mit PFI

Tab. 2 gibt einen Überblick über die Absolutwerte der prä- und postoperativen CCA in allen drei Kniestellungen bei Patienten mit PFI sowohl in unbelasteten als auch in belasteten Situationen. Im Vergleich der CCA bei Patienten mit PFI in verschiedenen Belastungssituationen ergeben sich keine signifikanten Unterschiede.

**Table 2:** 

		CCA ohne Quadrizepsaktivierung (in mm^2^)	CCA mit Quadrizepsaktivierung (50 N, in mm^2^)	
Flexion	*n* =			*p* =
*Patienten mit PFI präoperativ*				
0°	15	67,3 ± 47,3	72,4 ± 45,9	0,820
15°	15	118,9 ± 56,6	112,5 ± 54,9	0,650
30°	15	267,6 ± 96,1	286,1 ± 92,7	0,064
*Patienten mit PFI postoperativ*				
0°	15	159,3 ± 51,4	141,0 ± 63,8	0,196
15°	15	189,3 ± 62,2	206,6 ± 67,7	0,328
30°	15	347,3 ± 52,1	353,5 ± 64,6	0,552
*Probanden mit gesunden Kniegelenken*				
0°	15	130,3 ± 66,0	130,7 ± 68,4	0,820
15°	15	191,5 ± 89,3	192,6 ± 87,7	0,156
30°	15	370,7 ± 91,9	409,0 ± 73,8	*0,003*

#### Patienten postoperativ nach MPFL-Plastik

**Figure 3:**
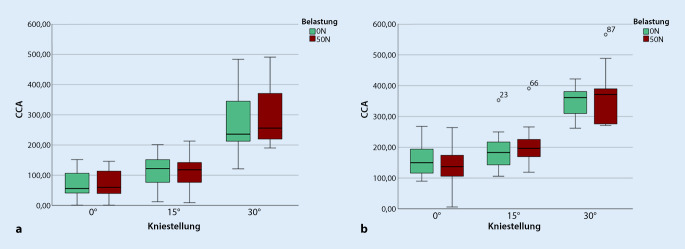

Bei patellofemoral instabilen Patienten ergeben sich postoperativ ebenso keine signifikanten Unterschiede der CCA in allen drei Flexionsgraden mit und ohne Quadrizepsaktivierung (Tab. 2). Abb. 3 visualisiert den Vergleich der CCA mit und ohne Belastung präoperativ (a) und postoperativ (b) anhand von Boxplots.

#### Probanden mit gesunden Kniegelenken

**Figure 4:**
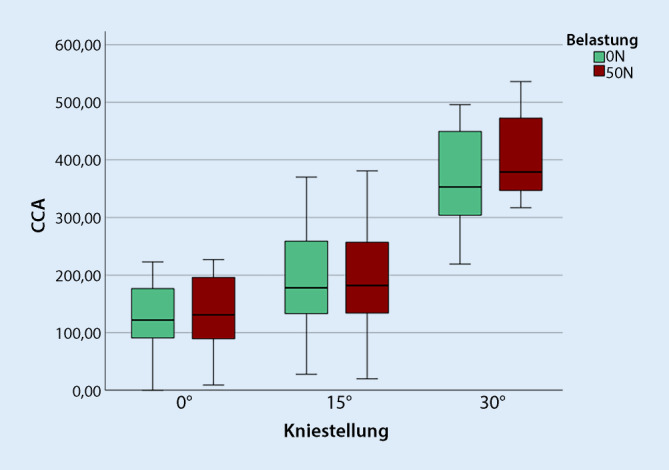

Auch bei kniegesunden Probanden ergeben sich im Vergleich der patellofemoralen CCA keine signifikanten Unterschiede sowohl in Streckung als auch in 15°-Flexion, jedoch zeigt sich eine signifikant vergrößerte CCA (Zuwachs von 10,3 %) in 30° Beugung (*p* = 0,003). Abb. 4 visualisiert den Vergleich der unbelasteten und belasteten (50 N) CCA der Probanden mit gesunden Kniegelenken.

Im Vergleich der CCA über die Zunahme der Beugung zeigen sich sowohl bei den Patienten präoperativ als auch postoperativ, wie auch bei den gesunden Volontären von Streckung auf 15° Beugung (*p* = 0,001; *p* = 0,028; *p* = 0,005) sowie von 15° Beugung auf 30° Beugung (*p* = 0,001; *p* = 0,001; *p* = 0,001) statistisch signifikante Unterschiede.

Auch unter Belastung fallen diese Unterschiede statistisch signifikant aus, sowohl von Streckung auf 15° Beugung (*p* = 0,001; *p* = 0,004; *p* = 0,001) als auch von 15° Beugung auf 30° Beugung (*p* = 0,001; *p* = 0,003; *p* = 0,001), präoperativ wie postoperativ als auch bei kniegesunden Probanden.

In allen drei Positionen kann sowohl in unbelasteten als auch in belasteten Situationen ein Zuwachs der CCA prä- zu postoperativ beobachtet werden. In Streckung zeigt sich dieser unbelastet bei *p* = 0,001 und belastet bei *p* = 0,013, in 15° Beugung bei *p* = 0,013 bzw. *p* = 0,002 und bei 30° Beugung bei *p* = 0,005 bzw. mit Quadrizeps Aktivierung bei *p* = 0,013.

## Diskussion

Das wichtigste Ergebnis der vorliegenden Studie ist, dass Patienten mit strecknaher patellofemoraler Instabilität keinen relevanten Einfluss der Quadrizepsaktivierung auf die patellofemoralen CCA, weder bei 0°, 15° noch bei 30° Flexion, aufweisen. Auch nach einer patellofemoralen Stabilisierung mittels MPFL-Plastik zeigt sich unter Quadrizepsaktivierung keine signifikante Änderung der patellofemoralen CCA.

Der stabilisierende Einfluss der Muskulatur auf das patellofemorale Tracking steigt bei gesunden Kniegelenken im Verlauf der frühen Kniebeugung an und erreicht sein Maximum bei etwa 30° Flexion [3]. Diese Beobachtung konnte im Rahmen der vorliegenden Auswertung weiter gestützt werden. In der kniegesunden Studienpopulation zeigt sich in 30° Beugung ein durchschnittlicher, statistisch signifikanter Anstieg der CCA um 10,3 % im Vergleich mit und ohne aktivierter Quadrizepsmuskulatur; bei Patienten mit PFI hingegen zeigt sich in allen drei untersuchten Flexionsgraden kein signifikanter Unterschied der CCA während der Muskelaktivierung. Im Rahmen dieses Forschungsprojektes konnte anhand eines Vergleiches kinematischer Parameter zwischen kniegesunden und patellofemoral instabilen Patienten (*n* = 17/17) diese Beobachtung, mit einem durchschnittlichen Zuwachs der CCA bei gesunden Probanden um 11,2 % in 30° Beugung, ebenso aufgezeigt werden [24].

Als ein Standbein der primären Behandlung klinisch manifestierter patellofemoraler Instabilitäten sowie der Behandlung von Patellaerstluxationen wird die konservative Therapie angesehen, welche unter anderem aus der Kräftigung der potenziell patellofemoral stabilisierend wirkenden Muskelgruppen (z. B. M. vastus medialis) besteht [18, 25]. Anhand der Ergebnisse des vorliegenden Vergleiches zeigt sich bei Patienten mit PFI weder in Streckstellung noch in 15° oder 30° Beugung ein signifikanter Einfluss der Quadrizepsmuskulatur auf die patellofemorale CCA. Entgegen der Einschätzung, dass Menschen mit gesunden Kniegelenken einen stabilisierenden Einfluss der Quadrizepsmuskulatur auf die patellofemorale Artikulation mit einer einhergehenden Vergrößerung der patellofemoralen CCA ab etwa 30° Beugung aufweisen, kann dies zumindest im Durchschnitt bei Patienten mit PFI nicht festgestellt werden. Die Ursachen dieser Beobachtung sind bislang nicht geklärt. Mögliche Einflussfaktoren könnten durch veränderte Kraftvektoren der Quadrizepsmuskulatur auf die Kniescheibe, durch Unterschiede des muskulären Trainingszustandes kniegelenksführender Muskelgruppen, durch Unterschiede der ossären Geometrie sowie durch strukturelle Gewebsunterschiede erklärt werden. Um individuelle Unterschiede der Maximalkraft zu minimieren, wurde für das vorliegende Studiendesign eine standardisierte Quadrizepsaktivierung mit 50 N genutzt, um lediglich eine Aktivierung der Muskulatur zu erzielen.

Anhand dieser Beobachtungen ist der grundsätzliche Stellenwert der konservativen Therapie bei Patienten mit strecknaher PFI, insbesondere der Stellenwert des isolierten Muskelaufbaus patellofemoral stabilisierend wirkender Muskelgruppen, z. B. mittels Elektrostimulation oder EMG-Biofeedback [18], mit dem Ziel der Wiederherstellung/Verbesserung der patellofemoralen Kontaktfläche in ihrer Effektivität infrage zu stellen bzw. sind Studien mit dem Nachweis der Effektivität zu fordern.

Bei zwei Patienten der vorliegenden Studienpopulation zeigte sich allerdings eine CCA-Vergrößerung in 30° Flexion mit einem Anstieg (jeweils > 10 %), der vergleichbar mit kniegesunden Probanden ist. Ob sich diese Patienten besonders für einen konservativen Therapieversuch eignen, gilt es weiter zu beobachten bzw. Patientengruppen besser zu identifizieren, die von einer Modifikation der Muskelaktivität profitieren. Gemäß unserer Einschätzung bedarf es diesbezüglich weiterer prospektiver Kohortenstudien zur Bestimmung und Evaluation weiterer Faktoren, um den Erfolg der konservativen Therapie abschätzen zu können und Subjekte zu identifizieren, welche von einer primär operativen patellofemoral stabilisierenden Therapie, z. B. mittels MPFL-Plastik, profitieren würden.

Die Rekonstruktion des MPFL hat sich zu einem weit verbreiteten chirurgischen Verfahren bei rezidivierenden Patellaluxationen entwickelt. Ziel der MPFL-Rekonstruktion ist es, die Stabilität des PFJ wiederherzustellen und die patellofemorale Kinematik sowie den Kontaktmechanismus zu normalisieren, was zur Linderung klinischer Symptome und zum Erhalt des Gelenks führt [1, 5]. Die Verbesserung der patellofemoralen CCA kann daher als ein Kernziel dieses Verfahrens angesehen werden. Kürzlich konnte anhand verschiedener Studien gezeigt werden, dass patellofemoral stabilisierende Operationen zu einer Zunahme der patellofemoralen CCA und damit zur Verbesserung der patellofemoralen Kongruenz führen [14].

Eine weitgehende Normalisierung der patellofemoralen CCA durch eine patellofemorale Stabilisierung mittels MPFL-Plastik konnte bereits in einer gesonderten Publikation des vorliegenden Forschungsprojektes festgestellt werden [16]. Diese Beobachtung konnte ebenso durch die vorliegende Auswertung bestätigt werden. Die Ergebnisse der vorliegenden Studie zeigen nun jedoch erstmals in vivo anhand einer statisch-dynamischen MRT-Studie, dass Patienten mit strecknaher PFI auch nach einer Stabilisierung mittels MPFL-Plastik keinen signifikanten Einfluss der Quadrizepsmuskulatur im Bereich der frühen Kniebeugung (0–30°) auf die patellofemorale CCA aufweisen. Zwar zeigt sich eine signifikant vergrößerte patellofemorale Kontaktfläche nach MPFL-Plastik und somit zu vorherigen Studien konsistente Ergebnisse, die Wiederherstellung einer physiologischen Gelenkkinematik, als Zusammenspiel der passiven (weichteilige/ligamentäre Strukturen sowie ossäre Geometrie) und der aktiven Stabilisatoren (muskuläre Kniegelenksführung) kann folglich allerdings allein durch eine MPFL-Rekonstruktion nicht erreicht werden. Eine postoperative Medialisierung der Patella durch die MPFL-Plastik führt erwartbar zusätzlich zu einer geminderten Vorspannung etwaiger medialisierend/stabilisierend wirkenden Muskelanteilen des Quadrizeps und folglich zu dieser Beobachtung. Der MPFL-Plastik als patellofemoraler Stabilisator, sowie konsekutiv der Integration des MPFL-Transplantates im (frühen) postoperativen Verlauf, kommt daher als primärem Stabilisator der Patella im Rahmen der strecknahen patellofemoralen Artikulation (0–30°) aufgrund einer ausbleibenden zusätzlichen muskulären Stabilisierung eine essenzielle Rolle zu.

MRT-Untersuchungen haben sich in letzter Zeit zu einem wichtigen Werkzeug für die In-vivo-Beurteilung des patellofemoralen Gelenks entwickelt [26, 27]. Das patellofemorale Maltracking kann somit während der frühen Beugung dynamisch anhand von In-vivo-MRT quantifiziert werden. Es besteht nach wie vor Bedarf an weiteren hochauflösenden MRT-in-vivo-Studien, um Faktoren des patellofemoralen Maltrackings zu identifizieren, welche uns das Ziehen von Rückschlüssen bezüglich möglicher operativer Zusatzverfahren (z. B. Trochleaplastik, Tubersitasversatzosteotomien, Korrekturosteotomien) zur optimalen Behandlung patellofemoraler Instabilitäten erlauben. In der vorliegenden Studie wurde ein halbautomatisiertes Deep-Learning-Netzwerk verwendet, um die Knorpeloberfläche von Femur und Patella zu quantifizieren und die patellofemorale Knorpelkontaktfläche mithilfe von 3‑D-Meshes zu generieren [23]. Wie zuletzt im Rahmen einer Interrater-Auswertung in der Beurteilung von Trochleadysplasien, sowie auch für andere dreidimensionale Körper aufgrund ihrer Komplexität, gezeigt wurde, scheint die 3‑D-Analyse ein präzises Werkzeug für die Bestimmung von dreidimensionalen Strukturen und Oberflächen zu sein, welche zu exakten klinischen Bewertungen und folglich präzisen Diagnosen führt [28–30].

## Limitationen

Eine Einschränkung dieser Studie ist, dass sie nur eine kleine Anzahl von Fällen umfasste (*n* = 15/15). Die Ergebnisse sind daher als explorativ zu verstehen und können bei größeren Studienpopulationen anders ausfallen.

Einer der Nachteile der dynamischen MRT-Bildgebung, mit dem sich auch andere Studien befasst haben, ist das Problem der Bewegungsartefakte [22]. Um dieses Problem zu reduzieren, wurde in unserem Setup eine prospektive Bewegungskorrektur genutzt [20–22]. Es muss konzediert werden, dass die verwendete Tracking-Kamera und ihr begrenzter Fokalbereich den schon durch die Scannerbohrung limitierten Knieflexionswinkel weiter einschränken. In unserem Versuchsaufbau ließen sich Beugungswinkel bis 30° aber auch für große Probanden problemlos realisieren. Da das MPFL oberhalb von 30° Kniebeugung einen zunehmend geringeren Einfluss auf das Patella-Tracking zu haben scheint, wären größere Flexionswinkel für die Überprüfung der gegebenen Hypothese vermutlich von geringem Mehrwert. In dieser Studie wurde das CNN-Modell nur für die automatische Segmentierung der Knochen, nicht aber für die Knorpel trainiert. Während die automatisch segmentierten Knochenmasken nur indirekt für eine grobe Ausrichtung der Bilder verwendet wurden, bildeten die Knorpelmasken die Grundlage für die Berechnung der CCA bei jedem Beugewinkel, sodass eine wesentlich genauere Knorpelsegmentierung erforderlich war. Bei der vorhandenen Anzahl von Proben in der Datenbank konnte dieses Genauigkeitsniveau mit einem automatischen Segmentierungsmodell nicht erreicht werden. Um dieses Problem zu lösen, haben wir die beschriebene Knochenregistrierung verwendet, um die manuell segmentierten Knorpel vom Basisbild auf die Bilder des Kniegelenkes für 15 und 30° zu übertragen.

## Schlussfolgerung

Bei Patienten mit strecknaher patellofemoraler Instabilität zeigt sich kein signifikanter Einfluss der Quadrizepsmuskulatur auf die patellofemoralen CCA. Auch nach patellofemoraler Stabilisierung mittels MPFL-Plastik zeigt sich keine signifikante Änderung der CCA bei aktivierter Quadrizepsmuskulatur. Eine patellofemorale Stabilisierung mittels MPFL-Plastik kann im Einzelfall zu einer Normalisierung der CCA führen, nicht aber zu einer Normalisierung des patellofemoralen Kontaktmechanismus als Zusammenspiel aktiver und passiver patellofemoraler Stabilisatoren.

## Fazit für die Praxis

- Patienten mit strecknaher patellofemoraler Instabilität zeigen, anders als Kniegesunde, weder prä- noch postoperativ, einen signifikanten Einfluss einer Quadrizepsaktivierung auf den patellofemoralen Knorpelkontmechanismus.
- Eine MPFL(mediales patellofemorales Ligament)-Plastik bewirkte bei allen Patienten eine Steigerung der patellofemoralen Kontaktfläche im Rahmen der initialen Kniebeugung in allen gemessenen Positionen (0–30°).
- Eine komplette Normalisierung des Kontakts der patellofemoralen Kontaktfläche im Rahmen der initialen Kniebeugung konnte nur in einzelnen Patienten erreicht werden.
